# Exploring Longitudinal Associations Among Error-Related Negativity, Perception of Interpersonal Events, and Internalizing Symptoms in Female Adolescents

**DOI:** 10.1007/s10802-026-01485-4

**Published:** 2026-07-15

**Authors:** Rebecca Mueller, Katie L. Burkhouse, Jennifer H. Suor

**Affiliations:** 1 Department of Psychology, The University of Texas at Austin, Austin, Texas USA; 2 Department of Psychology, The Pennsylvania State University, University Park, Pennsylvania USA; 3 Department of Psychiatry, University of Illinois Chicago, 11601 W. Taylor Street, Chicago, Illinois 60612 USA

**Keywords:** Error-related negativity, Adolescence, Internalizing symptoms, Appraisal bias, Interpersonal relationships

## Abstract

Heightened error-related negativity (ERN), a neural marker of error sensitivity and trait defensive reactivity, and maladaptive appraisals of interpersonal events have each been linked to adolescent internalizing symptoms. Few studies have evaluated a developmental mechanistic model to test whether enhanced ERN might prospectively predict more negative perceptions of peer and family events, and if through this pathway, internalizing symptoms increase. The present study tested this hypothesis in a sample of 79 female adolescents (ages 11–16 years old), of whom 36 adolescents had mothers with histories of internalizing disorders. At baseline, the participants’ ERN was measured through electroencephalogram recordings while completing a computerized flanker task. At baseline and 6-month follow-up, participants completed self-report measures of adolescent perceptions of family and peer events and internalizing symptoms. Analyses were conducted within a structural equation modeling framework to capitalize on the full dataset. Three competing models (correlated outcomes, negative perceptions of interpersonal events mediation, reverse internalizing symptoms mediation) were tested and relative model fit comparisons were made to determine which model fit the data better. We found partial support for our hypotheses. For the family events models, there was stronger evidence that baseline ERN predicted increases in internalizing symptoms via increases in negative perceptions of family events. However, for the peer events models, there was stronger evidence that internalizing symptoms mediated the prospective associations between baseline ERN and increases in negative perceptions of peer events. Future research should consider reciprocal effects among these pathways and how these processes could be targeted by preventive interventions.

**Supplementary Information:**

The online version contains supplementary material available at 10.1007/s10802-026-01485-4.

## Introduction

Internalizing symptoms broadly refer to psychological distress and negative emotional experiences that are directed inward (Zahn-Waxler et al., 2000). These symptoms often manifest as persistent feelings of sadness, worry, fear, insecurity, guilt, self-blame, and shame, and are strongly associated with internalizing psychopathologies such as depressive disorders and anxiety disorders (APA, 2013). Internalizing symptoms frequently increase in adolescence, which is a critical developmental period characterized by heightened emotional reactivity and vulnerability (Costello et al., 2003; Kessler et al., 2005). The average age of onset of internalizing symptoms and disorders is 14 years old (Kessler et al., 2005). Epidemiological data indicate that by age 18, approximately one-third of adolescents will have met criteria for an anxiety disorder and about 14% will have met criteria for a mood disorder (Merikangas et al., 2010), with individuals assigned female sex at birth at greater risk compared to male adolescents (Shorey et al., 2022). Adolescence is a higher risk period for the emergence of psychopathology, with internalizing symptoms being the most prominent in female adolescents (Zahn-Waxler et al., 2000). Age-related changes in multiple neural systems implicated in motivational and executive control processes influence how adolescents interpret their worlds and interact with family members and peers (Lichenstein et al., 2016; Somerville et al., 2006). Yet little is known about how individual differences in neural risk markers for sensitivity to negative environmental cues might be prospectively associated with increases in negative appraisals of interpersonal experiences, and in turn, may be associated with increases in internalizing symptoms that are typically observed during this developmental period. To this end, the present study tests a multifactorial longitudinal model to evaluate associations among neural mechanisms implicated in subjective negative appraisals of environmental cues and internalizing psychopathology in a sample of female adolescents.

### Error-Related Negativity (ERN) and Internalizing Disorders

The error-related negativity (ERN), an event-related potential component, is one well-established neural marker of performance monitoring after making errors and trait defensive reactivity, and it has been associated with internalizing psychopathologies (e.g., Härpfer & Riesel, 2026; Weinberg et al., 2012). The ERN is measured from electroencephalogram (EEG) recordings while an individual completes an error monitoring task. The ERN is a negative deflection that occurs in the waveform 0-100 ms following the commission of errors and is generated by activity in the anterior cingulate cortex (ACC; Dehaene et al., 1994; Holroyd & Coles, 2002).

Greater ERN amplitudes are considered to reflect a greater sensitivity to making errors and heightened defensive reactivity to negative environmental cues (e.g., Olvet & Hajcak, 2008; Weinberg et al., 2012). Increasing evidence suggests the ERN could be a potential endophenotypic biomarker of internalizing psychopathology risk (Moser et al., 2018; Suor et al., 2022) as it satisfies most of the criteria for being classified as an endophenotypic biological marker of internalizing psychopathology, particularly anxiety (Gottesman & Gould, 2003). Specifically, the ERN demonstrates trait-like consistency and high test-retest reliability (Meyer et al., 2014), and demonstrates familial co-segregation as indicated by concordance between first-degree relatives (Burwell et al., 2016; Meyer et al., 2012). Additionally, an enhanced ERN has been observed among healthy first-degree relatives of patients with anxiety disorders (Anokhin et al., 2008; Moser et al., 2018) and prospectively predicts the onset of anxiety disorders (Meyer et al., 2015, 2018).

With respect to diagnostic specificity of associations between ERN and different internalizing psychopathologies, amplified ERNs and increases in anxiety symptoms have been documented in preschool and school-age children, with research demonstrating that early ERN profiles predict anxiety symptom trajectories (Meyer, 2017). Studies have also documented enhanced ERN profiles among patients with anxiety, such as generalized anxiety disorder, social anxiety disorder, and obsessive-compulsive disorder (Hajcak et al., 2003). Findings regarding associations between ERN and depression symptoms are less consistent and may depend on specific symptom dimensions or comorbidity with anxiety (Weinberg et al., 2016; Moser et al., 2013). While some studies have identified a blunted ERN in individuals with depressive symptoms—suggesting disengagement from monitoring of performance errors (Olvet & Hajcak, 2008)—others have reported enhanced ERN responses, particularly in individuals with comorbid anxiety, moderate depressive symptom severity, and high levels of self-critical thinking (Olvet & Hajcak, 2008; Weinberg et al., 2015).

Given this mixed evidence, along with high levels of anxiety and depressive disorder comorbidity, recent evidence indicates it could be helpful to examine clusters of personality traits related to individual differences in ERN patterns (Hill et al., 2016; Olvet & Hajcak, 2008; Ren et al., 2025). Some studies have found that a heightened ERN is more generally associated with personality traits of negative affect, neuroticism, and negative biases and appraisal of social cues, which are traits that are commonly present across different anxiety and depressive disorder clinical presentations (Olvet & Hajcak, 2008). For example, Hill and colleagues (2016) found that ERN amplitudes were associated with personality traits, such that individuals characterized by lower conscientiousness and higher negative urgency exhibited larger ERN amplitudes, suggesting that personality profiles involving impulsivity and regulatory control may shape neural responses to errors. Recent integrative reviews have further highlighted that heightened ERN responses tend to be associated with traits related to threat sensitivity and negative affectivity, and reduced ERN amplitudes have been linked to impulsivity, risk-taking, and sensation-seeking tendencies (Härpfer & Riesel, 2026). Related to this line of inquiry, Ren and colleagues (2025) found that repetitive negative thinking, particularly brooding, qualifies the association between diagnostic groups and ERN profiles. Specifically, they found that individuals with comorbid anxiety and depression who also endorse repetitive negative thinking patterns demonstrated a more heightened ERN relative to individuals with depressive disorder diagnoses without anxiety comorbidities and with those who report engaging in less repetitive negative thinking patterns (Ren et al., 2025). One interpretation is that the ERN may reflect underlying ruminative and evaluative processes that shape how individuals appraise family and peer events, which could increase risk for internalizing symptoms, or they could mutually influence each other over time. Integrating recent personality and social cognition perspectives on ERN within a developmental framework, the current study sought to evaluate associations among heightened ERN, perceptions of common family and peer events, and internalizing symptoms in female adolescents within a short-term follow-up design.

### Interpersonal Events and Internalizing Disorders

In addition to biological risk markers, a substantial body of work has focused on how interpersonal stressors and events contribute to internalizing disorders during adolescence (Hankin et al., 2007; Compas et al., 2017). For example, stressful life events, such as parental divorce, bereavement, or significant disruptions in living conditions, have been shown to predict greater emotional and behavioral maladjustment in children and adolescents (Grant et al., 2004; Compas et al., 2017). Other more developmentally normal events can also have a significant impact, particularly those occurring within family and peer environments. Moreover, normative events can be similarly emotionally salient as more stressful ones, and exert comparable effects on emotional functioning, due to their relational importance (Hankin et al., 2007; Rudolph et al., 2000).

Developmental theories of interpersonal stress emphasize that the sources and impact of social events change across childhood and adolescence (Rudolph et al., 2000; Grant et al., 2004; Compas et al., 2017). During early and middle childhood, family relationships serve as the primary context for both support and stress exposure (Rudolph et al., 2000; Sheeber et al., 2007). Family-related stress has been shown to predict internalizing symptoms across multiple developmental stages and may be particularly salient during early adolescence when youth remain strongly embedded in the family context (Rudolph et al., 2000; Sheeber et al., 2007). As youth transition and progress through adolescence, peers and romantic partners become central sources of emotional support and stress. Interpersonal difficulties in these domains such as peer rejection, friendship conflict, and romantic breakups are linked to heightened risk for mood and anxiety disorders (La Greca & Harrison, 2005; Starr & Davila, 2012). This is particularly the case for female adolescents who exhibit greater attunement and sensitivity to social cues (McClure, 2000), endorse greater needs for social connectedness and approval in peer relationships (Rudolph & Conley, 2005), and report greater internalizing symptoms. Importantly, prior work indicates that adolescents’ subjective appraisals of interpersonal events may be more robust predictors of internalizing symptoms than the mere occurrence of events (Eberhart & Hammen, 2006; Liu & Alloy, 2010; Shapero & Steinberg, 2013). Thus, our study sought to evaluate how an established neural risk marker for greater negative affectivity and appraisal biases might predict changes in subjective appraisals of family and peer events and adolescent internalizing symptoms over a short-term follow-up period, as well as if these patterns were still present after accounting for the number of interpersonal events adolescents experienced during this time frame.

### ERN and Appraisal of Interpersonal Events

Given that the ERN is conceptualized to reflect heightened sensitivity to performance errors and negative environmental cues, adolescents with an amplified ERN may be more likely to perceive interpersonal events with greater personal threat and subjective negativity, thereby increasing vulnerability to internalizing symptoms. This aligns with stress-cognitive vulnerability models (Calvete et al., 2013; Liu & Alloy, 2010) and social information processing frameworks (e.g., Crick & Dodge, 1994) that propose individual differences in information processing and appraisal styles impact emotion, behavior, and mental health outcomes (Silk et al., 2003; Gunther Moor et al., 2010). Indeed, some research has shown that ERN amplitudes, even when measured in nonsocial tasks, are associated with subjective appraisal styles and cognitive coping patterns such as brooding and rumination (Ren et al., 2025). This could reflect the mechanistic pathway from ERN to internalizing symptoms via maladaptive appraisal styles (McClure, 2000; Rudolph & Conley, 2005).

### The Mediating Role of Negative Appraisals of Family and Peer Events

Taking this line of inquiry a step further, the present study examined whether greater subjective negative appraisals of developmentally salient peer and family events mediated prospective associations between heightened ERN patterns and increases in internalizing symptoms in female adolescents over assessment occasions spaced 6 months apart. A few studies have examined relevant questions and provided some initial support for the possibility that negative appraisals could act as a mediator between enhanced ERN and increases in internalizing symptoms. Given that adolescence is a developmental period marked by both maturation of error-monitoring systems (Meyer et al., 2012; Burwell et al., 2016) and greater sensitivity to interpersonal stressors (Rudolph et al., 2000; La Greca & Harrison, 2005), heightened ERN amplitudes may increase susceptibility to interpreting peer and family events in more negative ways, thereby exacerbating risk for internalizing symptoms. However, studies have yet to simultaneously evaluate prospective and longitudinal associations among enhanced ERN, appraisals of developmentally salient interpersonal events, and internalizing symptoms within a single model in a vulnerable sample of female adolescents, which highlights the need for longitudinal research that can begin to test these potential mediational pathways.

### The Present Study

**Fig. 1:**
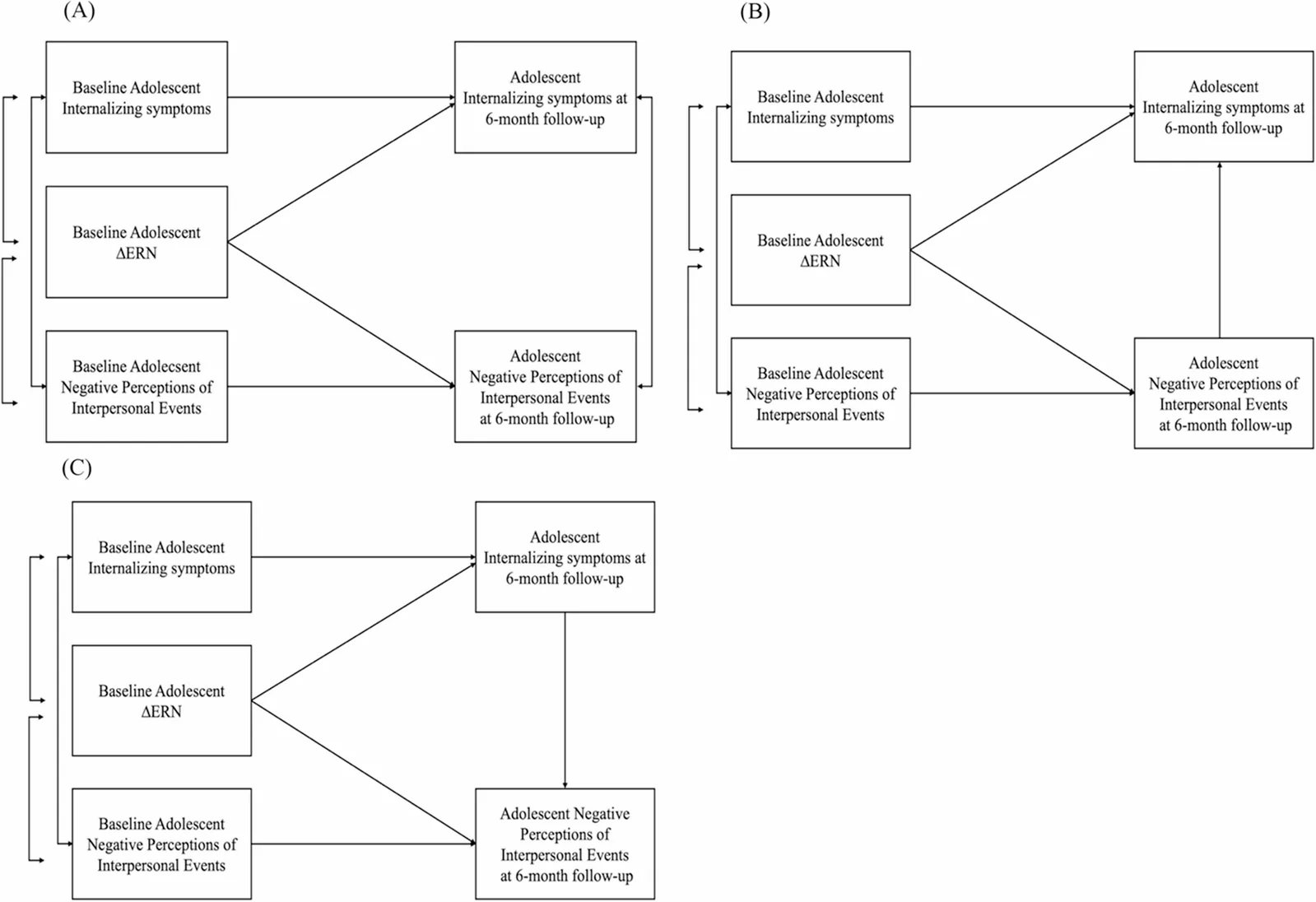
(**A**) Conceptual Figure of Correlated Outcomes Model. **(B)** Conceptual Figure of Negative Perceptions of Interpersonal Events Mediation Model. **(C)** Conceptual Figure of Internalizing Symptoms Mediation Model

In sum, the present study tested the hypothesis that greater ERN amplitude, an established neural correlate of internalizing psychopathologies and symptoms, prospectively predicted female adolescents’ changes in negative appraisals of recent family and peer interpersonal events over a 6-month period. Additionally, we tested the hypothesis that changes in negative appraisals of recent family and peer events would mediate prospective pathways from baseline amplified ERN to increases in internalizing symptoms from baseline to 6-month follow-up. We predicted that increases in adolescents’ negative perceptions of family and peer events would account for the indirect effect of enhanced ERN prospectively predicting increased internalizing symptoms from baseline to 6-month follow-up. Since we only had two waves of data where our proposed mediator and outcomes were measured at the same time point, we tested our proposed theoretical model against two competing models that could also account for the relationships among our variables: a correlated outcomes model, without directional pathways, and a reverse mediation model where internalizing symptoms mediated the prospective association between baseline enhanced ERN and increases in negative perceptions of family events (Fig. 1a-c). To test these hypotheses, we drew from an existing dataset that was designed to test brain-based mechanistic pathways from maternal depression to offspring who were assigned female at birth. Given the larger study design, the sample was well positioned to obtain variability in internalizing symptoms as approximately half of the female adolescents had a biological mother with a history of a depressive disorder, a well-established risk factor for internalizing symptoms given the high rates of intergenerational transmission of depression (Goodman, 2020), whereas the other half of female adolescents had biological mothers with no lifetime history of psychiatric illnesses.

## Methods

### Participants and Procedures

The sample consisted of 79 female adolescents (assigned at birth) between 11 and 16 years of age (*M*_*age*_ = 13.67, *SD* = 1.67) and their biological mothers. Dyads were recruited from a large metropolitan area in the Midwest through community outreach events, flyers, and online platforms (e.g., Facebook). Participants from the current study were drawn from a larger parent study that aimed to examine the intergenerational transmission of maternal depression, which had followed a two-group study design recruitment approach. The parent study recruited a group of dyads that were considered high-risk because their mothers had histories of major depressive disorders (MDD). The other recruited group was considered low risk as mothers of the female adolescents did not have any lifetime histories of psychiatric illnesses. Maternal diagnostic status was confirmed using the Structured Clinical Interview for DSM-IV Axis I Disorders (SCID-I; First, 2015). Thirty-six female adolescents had mothers with histories of MDD, and 43 female adolescents had mothers without histories of psychiatric illness. Several mothers with histories of depression also had histories (lifetime/current combined) of panic disorder (*n* = 2), agoraphobia (*n* = 2), obsessive compulsive disorder (*n* = 1), specific phobia (*n* = 1), social anxiety (*n* = 3), and generalized anxiety disorders (*n* = 6). At baseline, female adolescent psychiatric histories were assessed with the Kiddie Schedule for Affective Disorders and Schizophrenia for School-Age Children – Present and Lifetime Version (KSADS-PL; Kaufman et al., 2016). Across groups, female adolescents could not meet criteria for lifetime or current depressive disorders. With both the high and low risk group, some female adolescents had current or past anxiety disorders and attention deficit hyperactivity disorder, although the group of adolescents with maternal histories of MDD had the largest proportion of psychiatric disorder histories (*n* = 11). Despite this, there were no statistically significant differences across the different psychiatric disorders between groups. See Table 1 for female adolescents’ diagnoses across groups. Mothers and female adolescents were excluded if they met criteria for (within 6 months) alcohol or substance dependence, a lifetime diagnosis of bipolar disorder, schizophrenia, intellectual disability, serious medical illness, pervasive developmental disorders, or current suicidal ideation.

**Table 1:** Descriptive characteristics across groups

	LR Group (*n* = 43)		MDD Group (*n* = 36)	Overall (*n* = 79)				
	M	SD	M	SD	M	SD	F	*p* value
Base. Adolescent Age	13.88	1.72	13.42	1.59	13.67	1.67	1.54	0.218
Base. Neg. Percept. of Peer Events	-2.76	3.74	-2.47	3.33	-2.63	3.54	0.12	0.731
Base. Neg. Percept. of Family Events	-2.24	4.36	-3.35	4.62	-2.75	4.48	1.14	0.289
6 mos. Neg. Percept. of Peer Events	-2.16	3.12	-3.44	4.42	-2.69	3.74	1.58	0.214
6 mos. Neg. Percept. of Family Events	-2.47	3.44	-3.22	4.28	-2.78	3.12	0.52	0.476
Base. Total Peer Events	6.24	3.06	6.79	2.53	6.49	2.83	0.70	0.406
Base. Total Family Events	6.98	3.02	7.50	2.84	7.21	2.93	0.59	0.445
6 mos. Total Peer Events	6.22	1.93	6.44	2.48	6.31	2.16	0.13	0.718
6 mos. Total Family Events	6.84	2.54	6.65	2.21	6.76	2.39	0.09	0.772
Base. Internalizing Symptoms	11.35	6.83	13.56	7.43	12.35	7.43	1.89	0.174
6 mos. Internalizing Symptoms	9.82	6.91	17.21	10.32	12.93	9.20	9.28	0.004
Accuracy (% Correct)	0.86	0.07	0.86	0.09	0.86	0.08	0.01	0.942
Total Errors	46.27	21.82	45.77	29.37	46.04	25.36	0.01	0.942
Avg. RT Error Trials (ms)	329.26	130.41	331.60	63.85	330.34	104.03	0.01	0.934
Avg. RT Correct Trials (ms)	449.93	128.17	477.35	107.61	462.66	118.81	0.74	0.394

	*N*	%	*N*	%	*N*	%	χ^2^	*p* value
Ethnicity							0.48	0.273
Hispanic	14	32.60	15	41.70	29	36.70		
Race							5.94	0.202
White	26	60.50	18	50.00	44	55.70		
African American	8	18.60	5	13.90	13	16.50		
Asian	5	11.60	2	5.60	7	8.90		
Multiracial	1	2.30	4	11.10	5	6.30		
Other/Unknown	3	7.00	7	19.40	10	12.70		
Adolescent Diagnoses (current/past)						Fischer’s Exact Test		
Social Anxiety, current	1	1.3%	2	2.5%	3	3.8%		0.59
Generalized Anxiety Disorder, current	2	2.5%	6	7.6%	8	10.1%		0.13
Generalized Anxiety Disorder, past	0	0.0%	1	1.3%	1	1.3%		0.46
Obsessive Compulsive Disorder, past	0	0.0%	1	1.3%	1	1.3%		0.46
Unspecified Anxiety Disorder, past	1	1.3%	0	0%	1	1.3%	.	1.00
Attention Deficit Hyperactivity Disorder	0	0.0%	2	2.5%	2	2.5%		0.20

*Base *Baseline, *6 mos *6-month Follow-up, *Neg *Negative, *Percept *Perception, *Avg *Average, *RT *Response Time

In terms of the racial and ethnic breakdown of the sample, 55.7% of female adolescents identified as White, while 16.5% identified as African American, 8.9% identified as Asian, 5.1% identified as Multiracial, and 13.9% reported other or unknown racial backgrounds. Additionally, 36.7% of the female adolescents identified as Hispanic/Latinx. The median annual family income was $105,001 - $110,000. Adolescents participated in an in-person baseline assessment where they completed self-report surveys assessing exposure to interpersonal events within the past 6 months and their subjective appraisals of these events and internalizing symptoms, along with a computerized EEG task to assess error monitoring. Six months after their baseline assessment, adolescents completed the same self-report surveys. The retention rate from baseline to 6-month follow-up was approximately 73.40%. Attrition was not associated with adolescent age, internalizing symptoms, or whether mothers did or did not have histories of MDD, race, or ethnicity.

All procedures were approved by the University of Illinois Chicago’s Institutional Review Board prior to data collection. Research staff obtained written informed consent and verbal assent from mothers and adolescents prior to their study participation and administration of the study protocol.

### Measures

#### Flanker Task

At baseline, participants completed a computerized Flanker task (Kujawa et al., 2016; Meyer et al., 2014) designed to assess performance monitoring in response to errors. This task has been shown to reliably elicit the ERN in both youth and adults and demonstrates excellent test-retest reliability (Foti et al., 2013; Olvet & Hajcak, 2009). The task includes a practice block of 30 trials, followed by 11 blocks of 30 test trials. During each trial, a row of five horizontally aligned arrowheads was presented on a computer screen for 200 ms, followed by an intertrial interval between 2,300 and 2,800 ms. On half of the trials, the arrowheads were congruent (>>>>> ), and on the remaining trials, the center arrowhead was incongruent with the flanking arrowheads (<<><< ). Participants were instructed to indicate the direction of the center arrowhead by pressing the left or right mouse button. To ensure enough error trials, performance-based feedback was provided at the end of each block. If accuracy was below 75%, the message “Please try to be more accurate” was displayed; if accuracy exceeded 90%, the message “Please try to respond faster” appeared. Otherwise, participants saw the message “You’re doing a great job.” Useable EEG data was based on the following criteria: error ≥ 6 and correct ≥ 6, response rates ≥ 6, an overall accuracy of at least 60%, and ≥ 6 useable EEG segments on error and correct segments after artifact rejection. EEG data was excluded if these criteria were not met (*n* = 12). Other reasons for missing EEG data were due to equipment failure/too much noise in the signal (*n* = 5) or participants did not complete the flanker task (*n* = 6). In total, 56 out of the 79 participants had EEG data that was included in the analyses. Among these 56 participants, on average, adolescents committed 46.04 errors (*SD* = 25.36), which corresponds to an overall average accuracy of 86.00% (*SD* = 0.08). The average response time across correct and error trials was 462.66 ms (*SD* = 118.81) and 330.34 ms (*SD* = 104.03), respectively. The average reaction time on incorrect trials was faster compared to correct trials (*t* (56) = 13.04, *p* < .001).

### EEG Data Acquisition and Preprocessing

Continuous EEG data were recorded during completion of the Flanker Task using the BioSemi (Amsterdam, Netherlands) 34-channel cap (32 channels plus FCz and lz). Additional electrodes were placed on the right and left mastoids. Vertical and horizontal electrooculogram (EOG) activity was monitored to detect ocular artifacts (e.g., resulting from eye blinks): two electrodes were positioned 1 cm above and below the right eye to measure vertical eye blinks and movements, and two electrodes were placed 1 cm lateral to the outer canthi to record horizontal eye movements. EEG data was digitized at 24-bit resolution with Least Significant Bit (LSB) value of 31.25 nV and a sampling rate of 1,024 Hz.

Offline preprocessing was conducted in Brain Vision Analyzer 2 software (Brain Products, Gilching, Germany). Data were converted to an average mastoid reference, filtered with high-pass and low-pass filters of 0.1 and 30 Hz, respectively, and segmented 500 ms before the response through 1,000 ms after the response. Eye blinks were corrected using the established Gratton et al. (1983) correction procedure. Semi-automated artifact rejection procedures were employed to identify and eliminate artifacts with a voltage step of > 50µV between sample points or a maximum voltage difference of 175 µV within 400 ms intervals. Additional artifacts were removed using visual inspection.

**Fig. 2:**
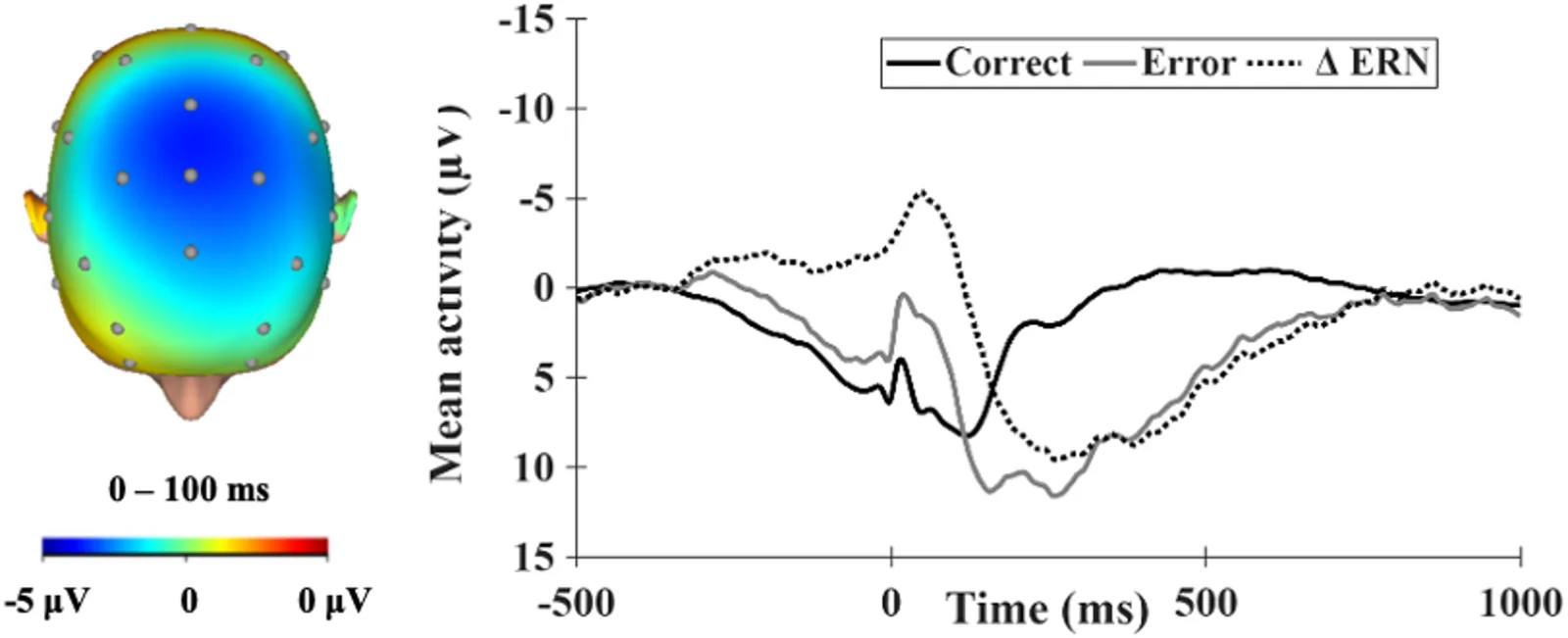
The topographical map of neural activity (error minus correct) and response-locked event-related potential waveforms at a pooling of Fz, FCz, Cz 0 − 100 ms after the response are shown. The raw waveforms for error and correct responses and raw difference score (error-correct) for ∆ERN (error-related negativity [ERN]; dotted line) are shown in the graph

Event-related potentials (ERPs) were averaged separately for error and correct trials and baseline-corrected using the − 500 to -300 ms pre-response interval. Inspection of the topographic maps indicated that neural activity following responses was maximal at frontocentral sites (Fig. 2). Consistent with prior studies (Boen et al., 2022, for review; van Meel et al., 2012), correct and error responses were scored by pooling frontocentral sites (Fz, FCz, Cz) as mean activity 0 to 100 ms after the response. The ERP response was more negative following errors versus correct responses (*t* (56) = -6.84, *p* < .001). We used a residual scoring approach to operationalize the ERN, which is consistent with recent recommendations in the EEG field (Meyer et al., 2017). To isolate error-specific variance, the ERN was calculated by using a residual scoring approach, which involves regressing the pooled error response variable on the pooled correct response variable and saving the unstandardized residual score, which was the variable used in the analyses. The waveform reflects the raw correct, error, and residual ∆ERN values (Fig. 2, prior to adjusting for covariates).

#### Internalizing Symptoms

At baseline and 6-month follow-up, adolescents completed the Youth Self-Report (YSR; Achenbach, 1991), a widely used, standardized self-report questionnaire consisting of 112 items that assess emotional and behavioral functioning in youth aged 11 to 18. The YSR instructs respondents to rate each item on a 3-point Likert scale (0 = *not true*, 1 = *somewhat or sometimes true*, 2 = *very true or often true*) based on their experiences in the past 6 months. The internalizing broad band raw score was used to assess adolescent internalizing symptoms at each time point. The internalizing symptoms scale has strong internal consistency and adequate convergent and discriminant validity in clinical and non-clinical samples (Achenbach, 1991), and congruence with diagnoses of depression and anxiety disorders obtained from psychiatric interviews (Doyle et al., 2007). In the present sample, the internalizing symptoms scale demonstrated strong internal consistency at baseline (α = 0.85) and 6-month follow-up (α = 0.90). At baseline, the mean for internalizing symptoms was 12.35 (*SD* = 7.15, Range = 1–34). For the 6-month follow-up, the mean score was 12.94 (*SD* = 9.72, Range = 0–43). The average raw scores in the current sample are comparable to YSR norm-referenced scores for female adolescents in this age group (M = 12.90. *SD* = 8.50; Achenbach, 1991). Furthermore, at both time points there was sufficient variability in internalizing scores as about a third of the adolescents had raw scores that fell outside normal limits for their age (Achenbach, 1991). Baseline and 6-month follow-up internalizing symptom scores were significantly correlated with each other across time (*r* = .68, *p* < .001).

#### Adolescent Perceived Events Scale

At baseline and 6-month follow-up, adolescents completed the Adolescent Perceived Events Scale (APES; Compas et al., 1987) to assess their exposure to, and perceptions of, recent life events. The APES is a self-report measure consisting of a checklist of 90 specific events relevant to adolescent development and is designed to capture life events across multiple domains, including family, peer, academic, romantic, and social networks within the past 6 months. The current study focused on peer events (15 items, e.g., “Change in number of friends,” “Make new friends or lose friends,” “Fight with or problems with a friend”) and family events (16 items, e.g., “Serious argument with a parent,” “Parents wanting you to do something or be a certain way”). The survey first asks whether specific family or peer events happened in the past 6 months. If the adolescent gives a “yes” response to an event, they are then asked to provide desirability ratings of how “good” or “bad” the event was for them when it happened. Adolescents were instructed that good events are ones that are pleasant or make you feel happy, while bad events are ones that upset you or make you feel scared, sad, or angry. Adolescents responded on a 9-point Likert scale ranging from − 4 (extremely negative) to + 4 (extremely positive), with a rating of 0 indicating the event was experienced as “neither good or bad.” Negative ratings of family and peer events scales are calculated by summing the desirability ratings when family and peer events were rated by the adolescent in the − 4 to 0 range. Greater negative scores on each scale indicate that adolescents felt the event had a greater negative impact on them. Studies have demonstrated that desirability ratings of events demonstrate concurrent associations with adolescent reported psychological symptoms (e.g., depressive symptoms and anxiety symptoms, rs ranging − 0.18 to − 0.26), and strong test-retest reliability, ranging from 0.74 to 0.89 (Cheng, 1997; Compas et al., 1987).

For exposure to family and peer events, we created sum scores of family events and peer events that adolescents reported they experienced over the past 6 months at each measurement occasion. At baseline, the mean number of family events reported was 7.21 (*SD* = 2.93, Range = 0 − 15) with an average perceived negativity rating of -2.75 (*SD* = 4.48, Range = − 23–0). The mean number of peer events reported was 6.49 (*SD* = 2.83, Range = 2–11) with an average perceived negativity rating of -2.63 (*SD* = 3.54, Range = -14–0). At the 6-month follow-up, the mean number of family events experienced within the past 6 months was 6.68 (*SD* = 2.39, Range = 2–12) with an average perceived negativity rating of -2.78 (SD = 3.79, Range = -15–0). For total peer events at the 6-month follow-up, the mean was 6.31 (*SD* = 2.16, Range = 2–11), with an average perceived negativity rating of -2.85 (*SD* = 3.90, Range = -16–0). Baseline and 6-month perceived negativity ratings of family events were significantly correlated (*r* = .77, *p* < .001). Baseline and 6-month perceived negativity ratings of peer events were significantly correlated (*r* = .29, *p* = .036). At each time point, greater total peer and family events demonstrated moderate significant correlations with adolescents endorsing more negative perceptions of peer and family events (*r*s ranged from − 0.37 to − 0.59, *ps* ≤ 0.01). Given these correlations, we include total family events at baseline and 6-month follow-up as a covariate in all our analytical models.

### Data Analysis Plan

To test the proposed hypotheses, we conducted a series of mediation path analyses within a structural equation modeling framework using AMOS, Version 29.0 (Arbuckle, 2022). Missing data along the baseline and follow-up variables in the dataset ranged from 0% to 30.4%. We examined if there were any patterns to the missingness. We calculated Little’s MCAR test to look at univariate and multivariate patterns of missingness among the variables in the analysis. The results indicated that data were missing completely at random (MCAR) (*χ*^2^ (56) = 60.23, *p* = .325). However, Little’s MCAR test may not perform as well in small samples (Enders, 2022; Graham, 2009; Little, 2013). To further evaluate the missing data mechanism, we conducted logistic regression analyses predicting missing EEG data from baseline variables. We did not do this for the other variables at baseline since they had less than 1.0% of missing data. These analyses indicated that participants missing EEG data reported higher baseline internalizing symptoms compared to those without missing EEG data (*B* = 0.89, *SE* = 0.25, Wald = 12.91, *p* < .001), and as such EEG missingness was related to an observed variable and is consistent with Missing At Random (MAR) mechanism. We inspected the data for normality, and there were no major violations in normality. There were two outliers on negative perceptions of family events at baseline (> 3.5 *SD*s from the mean). We re-ran analyses setting the outliers to the next highest value in the data set (3 *SDs* from the mean), and the results were identical. Given this, we decided to retain the original values in the dataset. Because the data were normally distributed, data were missing at random, and less than 50% of the data was missing, we used Full Information Maximum Likelihood (FIML) to estimate the missing data because it produces less biased estimates under MAR compared to listwise deletion (Enders, 2022; Little, 2013). As such, this allowed us to retain the full sample of 79 female adolescents in the analyses.

Our models were arranged similarly across the different models: correlated outcomes, hypothesized mediator model (negative perceptions of interpersonal events), and reverse mediation model (internalizing symptoms). For each model, we set baseline ∆ERN as the predictor variable. We modeled two measurements of negative perceptions of interpersonal events and internalizing symptoms, which were collected at baseline and 6-month follow-up. Autoregressive paths from baseline assessments to 6-month assessments were included in all the tested models to account for the variance in the 6-month assessments that was explained by baseline scores on that measure. We also included maternal MDD history as another predictor variable given established links between maternal MDD history and internalizing symptoms in offspring (Goodman, 2020) and significant bivariate correlations between maternal MDD history and internalizing symptoms at 6-month follow-up. Adolescent age and total interpersonal events at baseline and 6-month follow-up were included in their respective models as covariates.

For the mediation models, the directional paths were reversed depending on the model tested. For each model we executed, we calculated three model fit indices. The chi-square statistic tests the null hypothesis that the overidentified model fits the data as well as the fully saturated model (Wheaton, 1987). A nonsignificant chi-square (*p* > .05) indicates the fit between the specified model and the data is not significantly worse than the fit between the saturated model and the data. The comparative fit index (CFI; Bentler, 1990), is a goodness-of-fit measure that compares the tested model to the fit of the independence model. CFI values above 0.90 suggest acceptable fit. The root-mean-square error of approximation (RMSEA; Steiger, 2016) is an absolute measure of fit based on the non-centrality parameter; values less than 0.08 suggest acceptable fit. To test the robustness of our hypotheses, we conducted two alternative models for each primary mediation model. To determine which was the best fitting model, we compared chi-square, CFI, and RMSEA fit indices across all the models. We also examined the Akaike Information Criterion (Akaike, 1974) which is a relative model fit index that accounts for model complexity. Across all fit indices, lower values indicate better model fit relative to other models. Fit indices and results for the competing models are presented in Supplemental Materials Tables 1 and 2. Lastly, to test the significance of indirect effects, we used The PRODCLIN method (MacKinnon et al., 2007) via the RMediation web applet (Tofighi & MacKinnon, 2011), which computes the 95% confidence interval for the indirect effect using the distribution of the product of the coefficients’ method.

## Results

### Descriptive Statistics

Tables 1 and 2 show descriptive statistics and correlations among the study variables. Across most of the demographic, performance, and clinical measures, there were no significant differences between female adolescents whose mothers did or did not have MDD histories. However, at the 6-month follow-up, female adolescents who had mothers with MDD histories reported higher levels of internalizing symptoms compared to adolescents whose mothers did not have MDD histories (*M* = 9.82, *SD* = 6.91; *F* = 3.25, *p* = .001). According to Cohen (1988, 1992) guidelines, we observed moderate correlations between baseline and 6-month assessments of internalizing symptoms (*r* = .68) and between baseline and 6-month assessments of negative perceptions of interpersonal events (*r*s ranged = 0.27 − 0.77, See Table 2). Baseline ΔERN demonstrated a small non-significant correlation with baseline internalizing symptoms (*r* = − .20) and was moderately significantly correlated with internalizing symptoms at the 6-month follow-up (*r* = − .40). We observed moderate significant correlations between adolescent age and total peer events at baseline (*r* = .37) and total peer events at 6-month follow-up (*r* = .35). Adolescent age exhibited small nonsignificant correlations with baseline ΔERN (*r* = − .16), perceptions of interpersonal events at baseline and 6-month follow-up (*r*s = − 0.05 − 0.20), and internalizing symptoms at baseline (*r* = .10) and 6-month follow-up (*r* = .06). We observed a modest nonsignificant correlation between adolescent age and baseline total family events (*r* = .22) and a minimal association with total family events at 6-month follow-up (*r* = .05). We also examined partial regression scatter plots between baseline adolescent ΔERN and negative perceptions of interpersonal events and internalizing symptoms at 6-month follow-up, while adjusting for baseline measures. The plots showed linear associations between baseline adolescent ΔERN and the outcome variables (Supplemental Materials Figs. 1–3).

**Table 2:** Bivariate correlations among primary variables in the models

Variable	1	2	3	4	5	6	7	8	9	10	11	12	13
1. Adolescent Age	-												
2. Maternal Depression History	− 0.14	-											
3. ∆ERN (residual score)	− 0.16	0.04	-										
4. Base. Neg. Percept. of Family Events	− 0.05	− 0.12	0.41**	-									
5. Base. Neg. Percept. of Peer Events	− 0.13	0.04	0.03	0.27*	-								
6. 6-mos. Neg. Percept. of Family Events	− 0.19	− 0.10	0.49**	0.77**	0.29*	-							
7. 6-mos. Neg. Percept. of Peer Events	− 0.20	− 0.17	0.41**	0.54**	0.29*	0.70**	-						
8. Base. Internalizing Symptoms	0.10	0.16	− 0.20	− 0.33**	− 0.19	− 0.51**	− 0.39**	-					
9. 6-mos. Internalizing Symptoms	0.06	0.40**	− 0.40**	− 0.50**	− 0.09	− 0.60**	0.52**	0.68**	-				
10. Base. Total Family Events	0.22†	0.09	− 0.17	− 0.44**	− 0.22†	− 0.25†	− 0.14	0.08	− 0.20	-			
11. Base. Total Peer Events	0.37**	0.10	0.02	− 0.37**	− 0.51**	− 0.08	− 0.23	0.11	− 0.06	0.57**	-		
12. 6-mos. Total Family Events	0.05	− 0.04	− 0.03	− 0.22	− 0.12	− 0.38**	− 0.25†	0.11	0.04	0.59**	0.22	-	
13. 6 mos. Total Peer Events	0.35*	0.05	− 0.22	− 0.42**	− 0.25†	− 0.40**	− 0.59**	0.17	0.13	0.36**	0.46**	0.43**	-
*N*	79	79	56	75	75	55	55	79	57	75	75	55	55

*Base* Baseline, *6 mos* 6-months, *∆ERN* Error-related negativity residual, *Neg* Negative, *Percept* Perception

† *p *≤ .06, *p < .05; **p < .01

### Negative Perceptions of Family Events Models

First, we examined a correlated outcomes model where baseline adolescent ∆ERN predicted negative perceptions of family events and internalizing symptoms at 6-months. Autoregressive paths were included between baseline and 6-month assessments of negative perceptions of family events and internalizing symptoms, respectively. We also included path estimates for total family events at baseline and 6-month follow-up to the negative perceptions of family events and internalizing symptoms assessments at 6-month follow-up. Additionally, we included predictive pathways from maternal depression history and adolescent age to negative perceptions of family events and internalizing symptoms assessments at 6-month follow-up. For the correlated outcome models, we allowed the residuals of negative perceptions of family events and internalizing symptoms at 6-month follow-up outcomes to correlate with each other. This model did not include any directional paths between negative perceptions of family events and internalizing symptoms. Predictors and covariates were allowed to correlate with each other at baseline. We did not include a correlation between maternal MDD history and female adolescent age because there are no theoretical reasons to correlate the two constructs, and they did not demonstrate bivariate correlations. The model fit was unacceptable (*χ*^2^ (3, *N* = 79) = 10.92, *p* = .01; RMSEA = 0.18, 90% CI [0.08, 0.31]; CFI = 0.95; AIC = 112.92). We examined the path estimates. There was strong temporal stability between baseline and 6-month internalizing symptoms (*β* = 0.52, *SE* = 0.10, *p* < .001) as well as baseline negative perceptions of family events and 6-month follow-up assessment (*β* = 0.77, *SE* = 0.10, *p* < .001). Baseline heightened ∆ERN prospectively predicted increases in negative perceptions of family events (*β* = 0.23, *SE* = 0.07, *p* = .002) and increases in internalizing symptoms at 6-month follow-up (*β* = − 0.30, *SE* = 0.19, *p* = .002). Baseline total family events were associated with negative perceptions of family events (*β* = 0.20, *SE* = 0.14, *p* = .043) and internalizing symptoms at 6-month follow-up (*β* = − 0.39, *SE* = 0.35, *p* = .001). Greater total family events at the 6-month follow-up were associated with more negative perceptions of family events at 6-month follow-up (*β* = − 0.32, *SE* = 0.15, *p* < .001) but was not associated with internalizing symptoms at 6-month follow-up (*β* = 0.18, *SE* = 0.39, *p* = .103). Maternal MDD history prospectively predicted greater internalizing symptoms at 6-month follow-up (*β* = 0.30, *SE* = 1.60, *p* = .001) but was not associated with negative perceptions of family events at 6-month follow-up (*β* = − 0.02, *SE* = 0.63, *p* = .767). Adolescent age was not associated with any of the outcome variables. See Supplemental Table 3 for results.

Given that in the correlated outcomes model baseline adolescent ∆ERN prospectively predicted internalizing symptoms at 6-month follow-up and negative perceptions of family events at 6-month follow-up while controlling for prior assessments, we proceeded to test the mediation model with negative perceptions of family events set as the mediator. Structurally, the model was like the correlated outcomes model with the exception that we specified a directional path from negative perceptions of family events at 6-month follow-up to internalizing symptoms at 6-month follow-up. The model fit the data well (χ^2^ (3, *N* = 79) = 3.60, *p* = .308, RMSEA = 0.05, 90% CI [0.00, 0.20], CFI = 1.00; AIC = 105.60) and fit was relatively better than the correlated outcomes model (See Supplemental Materials Table 1 for Model Fit Comparisons). Baseline adolescent heightened ∆ERN prospectively predicted increases in negative perceptions of family events (*β* = 0.21, *SE* = 0.07, *p* = .003) while controlling for prior assessments and total number of family events (baseline and 6-month follow-up) (*β* = 0.75, *SE* = 0.09, *p* < .001), and increases in negative perceptions of family events at 6-month follow-up was associated with increased internalizing symptoms at 6-month follow-up (*β* = − 0.42, *SE* = 0.25, *p* < .001), while controlling for stability between baseline and follow-up internalizing assessments (*β* = 0.43, *SE* = 0.10, *p* < .001). Maternal MDD history continued to prospectively predict increases in internalizing symptoms at 6-month follow-up in the mediation model (*β* = 0.26, *SE* = 1.45, *p* = .002). See Table 3 for results. The total model accounted for 78.5% (R^2^ = 0.785) of the variance in negative perceptions of family events at 6-month follow-up and 68.2% (R^2^ = 0.682) of the variance in internalizing symptoms at 6-month follow-up, suggesting that over half of the variance in both outcomes were explained by the variables in the model. Next, we examined the significance of the indirect effect using the RMediation program. The indirect effect was significant, such that increases in negative perceptions of family events mediated the prospective association between baseline ∆ERN and increases in internalizing symptoms at 6-month follow-up (*z* = − 0.18, *SE* = 0.08, 95% CI [ -0.36, -0.04]).

**Table 3:** Final Model: ∆ERN, Negative perceptions of family events, and internalizing symptoms

Mediation Model: Negative Perceptions of Family Events	β	B	SE	*p*
Base. intern. symptoms → intern. symptoms at 6 mos.	0.43	0.51	0.10	< 0.001
Base. neg. percept. of family events → neg. percept. of family events at 6 mos.	0.75	0.73	0.09	< 0.001
Base. ∆ERN → neg. percept. of family events at 6 mos.	0.21	0.21	0.07	0.003
Base. ∆ERN → intern. symptoms at 6 mos.	− 0.15	− 0.30	0.20	0.125
Neg. percept. of family events at 6 mos. → intern. symptoms at 6 mos.	− 0.42	− 0.85	0.25	< 0.001
Base. total family events → intern. symptoms at 6 mos.	− 0.39	-1.16	0.32	< 0.001
Base. total family events → neg. percept. of family events at 6 mos.	0.21	0.31	0.14	0.025
Total family events at 6 mos. → intern. symptoms at 6 mos.	0.06	0.21	0.38	0.570
Total family events at 6 mos. → neg. percept. of family events at 6 mos.	− 0.31	− 0.55	0.15	< 0.001
Mat. MDD history → intern. symptoms at 6 mos.	0.26	4.55	1.45	0.002
Mat. MDD history → neg. percept. of family events at 6 mos.	− 0.00	− 0.03	0.63	0.960
Adolescent age → intern. symptoms at 6 mos.	0.05	0.25	0.42	0.559
Adolescent age → neg. percept. of family events at 6 mos.	− 0.05	− 0.14	0.18	0.450

*Base* Baseline, *Intern* Internalizing, *6 Mos* 6-Months, *∆ERN *Error-Related Negativity Residual, *Neg* Negative, *Percept *Perceptions, *Mat* Maternal, *MDD *Major Depressive Disorder

In the last test, we conducted a reverse mediation model where internalizing symptoms at 6-month follow-up was set as the mediator and the outcome was negative perceptions of family events at 6-month follow-up. The other parts of the model were the same as the correlated outcomes and first mediation model. The model fit did not fit the data well and was a relatively worse fit than the prior model (*χ*^2^ (3, *N* = 79) = 8.81, *p* = .030, RMSEA = 0.16, 90% CI [0.04, 0.28], CFI = 0.96; AIC = 110.81). Although model fit was poor, for comparative purposes we examined the path estimates. Baseline heightened ∆ERN predicted increases in internalizing symptoms at 6-month follow-up (*β* = − 0.29, *SE* = 0.19, *p* = .003), which in turn predicted increases in negative perceptions of family events at 6-month follow-up (*β* = − 0.22, *SE* = 0.05, *p* = .023). The direct path from baseline adolescent heightened ∆ERN to negative perceptions of family events at 6-month follow-up remained significant (*β* = 0.17, *SE* = 0.07, *p* = .034). See Supplemental Table 3 for results. The total model accounted for 61.8% (R^2^ = 0.618) of the variance in internalizing symptoms at 6-month follow-up and 77.7% of the variance in negative perceptions of family events at 6-month follow-up (R^2^ = 0.777), which was less than the variance accounted for in the negative perceptions of family events mediator model. We proceeded to test the significance of the indirect effect of adolescent ∆ERN on increases in negative perceptions of family events at 6-month follow-up via increases in internalizing symptoms at 6-months using RMediation web applet (Tofighi & MacKinnon, 2011). The results revealed a significant indirect effect such that, changes in internalizing symptoms at 6-month follow-up partially mediated the effect of baseline adolescent enhanced ∆ERN in prospectively predicting increases in negative perceptions of family events at 6-month follow-up (*z* = 0.06, *SE* = 0.03, 95% CI [ 0.01, 0.13]). However, given the reverse mediation model had poor fit and relatively worse fit compared to negative perceptions of family events mediator model, we found preliminary evidence that changes in negative perceptions of interpersonal events acts as the mediator between baseline adolescent ∆ERN and changes in internalizing symptoms.^1^

### Negative Perceptions of Peer Events Models

We followed the same modeling procedures for the negative perceptions of peer events models. We first tested a correlated outcomes model to determine whether there were direct effects between baseline ∆ERN and internalizing symptoms and negative perceptions of peer events at 6-month follow-up while controlling for autoregressive paths from each time point, total peer events (baseline and 6-month follow-up), maternal MDD history, and adolescent age. The model fit was not adequate (χ^2^ (3) = 5.86, *p* = .12, RMSEA = 0.11, 90% CI [0, 0.24], CFI = 0.98, AIC = 107.86; See Supplemental Table 2). Baseline internalizing symptoms predicted internalizing symptoms at 6-month follow-up, demonstrating temporal stability (*β* = 0.49, *SE* = 0.11, *p* < .001), but baseline negative perceptions of peer events did not significantly predict negative perceptions of peer events at 6-month follow-up (*β* = 0.15, *SE* = 0.11, *p* = .140). However greater total peer events at 6-month follow-up, but not total peer events at baseline, was associated with more negative perceptions of peer events at 6-month follow-up (*β* = − 0.52, *SE* = 0.21, *p* < .001). Total peer events across time points were not associated with internalizing symptoms at 6-month follow up (*β*s ≤ − 0.160, *p*s ≥ 0.177). Baseline adolescent ∆ERN prospectively predicted increases in negative perceptions of peer events at 6-month follow-up (*β* = 0.25, *SE* = 0.09, *p* = .022) and increases in internalizing symptoms at 6-month follow-up (*β* = − 0.28, *SE* = 0.22, *p* = .012). See Supplemental Table 4.

Since baseline adolescent ∆ERN was prospectively predictive of both negative perceptions of peer events and internalizing symptoms at follow-up while controlling for baseline assessments of these measures, we proceeded to test the hypothesized mediation where changes in negative perceptions of peer events at 6-month follow-up would mediate the prospective association between baseline adolescent ∆ERN and increases in internalizing symptoms at 6-month follow-up. The structure of the model was similar to the correlated outcomes model except now we included a directional path from negative perceptions of peer events at 6-months to internalizing symptoms at 6-month follow-up.

The model fit was questionable but fit the data relatively better than the correlated outcomes model (*χ*^2^ (3, *N* = 79) = 5.34, *p* = .149; RMSEA = 0.10, 90% CI [0.00, 0.24]; CFI = 0.98; AIC = 107.37; See Supplemental Materials Table 2). The autoregressive path from baseline to 6-month internalizing symptoms was significant (*β* = 0.48, *SE* = 0.10, *p* < .001). The autoregressive path from baseline and 6-month follow-up negative perceptions of peer events was not significant (*β* = − 0.37, *SE* = 0.12, *p* = .097). The paths from baseline adolescent heightened ∆ERN prospectively predicting increases in negative perceptions of peer events at 6-month follow-up (*β* = 0.25, *SE* = 0.09, *p* = .022) and negative perceptions of peer events to internalizing symptoms at 6-month follow up (*β* = − 0.37, *SE* = 0.27, *p* = .002) were significant, such that baseline adolescent heightened ∆ERN predicted increases in negative perceptions of peer events, which in turn predicted increases in internalizing symptoms. The direct path from baseline adolescent ∆ERN and increases in internalizing symptoms at 6-month follow-up was not significant when the indirect path was included in the model (*β* = − 0.18, *SE* = 0.21, *p* = .101). Maternal MDD history prospectively predicted increases in internalizing symptoms at 6-month follow-up (*β* = 0.29, *SE* = 1.62, *p* = .002) but was not associated with increases in negative perceptions of peer events at 6-month follow-up (*β* = − 0.16, *SE* = 0.28, *p* = .162). Total peer events at 6-month follow-up were associated with negative perceptions of peer events at 6-months (*β* = − 0.52, *SE* = 0.21, *p* < .001). None of the other paths including the total peer event variables (baseline and 6-month follow-up) were significant. Adolescent age was not associated with negative perceptions of peer events or internalizing symptoms at 6-month follow-up. The overall model accounted for 47.60% (R^2^ = 0.476) of the variance in negative perceptions of peer events at 6-month follow-up and 61.70% (R^2^ = 0.476) of the variance in internalizing symptoms at 6-month follow-up, respectively, indicating the model accounted for substantial variance in both outcomes. Please see Table 4 for all results. Although the model fit was questionable, we still proceeded to test the significance of the indirect effect of baseline adolescent ∆ERN prospectively predicting internalizing symptoms at 6-months via increases in negative perceptions of peer events at 6-month follow-up. The results revealed greater changes in negative perceptions of peer events significantly mediated the prospective association between baseline adolescent enhanced ∆ERN on increases in internalizing symptoms at 6-month follow-up (*z* = − 0.18, *SE* = 0.10, 95% CI [-0.41, − 0.02]). See Supplemental Table 4 for full results.

**Table 4:** Final Model: ∆ERN, Negative perceptions of peer events, and internalizing symptoms

Reverse Mediation: Internalizing Symptoms	β	B	SE	*p*
Base. intern. symptoms → intern. symptoms at 6 mos.	0.54	0.66	0.11	< 0.001
Base. neg. percept. of peer events → neg. percept. of peer events at 6 mos.	0.13	0.13	0.10	0.197
Base. ∆ERN → neg. percept. of peer events at 6 mos.	0.09	0.08	0.09	0.349
Base. ∆ERN → intern. symptoms at 6 mos.	− 0.26	− 0.51	0.22	0.018
Intern. symptoms at 6 mos. → neg. percept. of peer events at 6 mos.	− 0.40	− 0.17	0.05	< 0.001
Base. total peer events → intern. symptoms at 6 mos.	− 0.16	− 0.48	0.36	0.179
Base. total peer events → neg. percept. of peer events at 6 mos.	0.04	0.05	0.17	0.753
Total peer events at 6 mos. → intern. symptoms at 6 mos.	− 0.00	− 0.02	0.45	0.972
Total peer events at 6 mos. → neg. percept. of peer events at 6 mos.	− 0.53	− 0.88	0.19	< 0.001
Mat. MDD history → intern. symptoms at 6 mos.	0.32	5.62	1.75	0.001
Mat. MDD history → neg. percept. of peer events at 6 mos.	0.04	0.28	0.86	0.755
Adolescent age → intern. symptoms at 6 mos.	0.09	0.46	0.55	0.398
Adolescent age → neg. percept. of peer events at 6 mos.	0.02	0.05	0.25	0.847

*Base *Baseline, *Intern *Internalizing, *Mos *Months, *∆ERN* Error-Related Negativity Residual, *Neg *Negative, *Percept *Perceptions, *Mat *Maternal, *MDD *Major Depressive Disorder

Lastly, we tested the reverse mediation model where internalizing symptoms at 6-month follow-up was set as the mediator. The model fit the data well, (*χ*^2^ (3, *N* = 79) = 1.98, *p* = .576; RMSEA = 0.00, 90% CI [0.00, 0.16], CFI = 1.00; AIC = 103.98; See Supplemental Table 2) and resulted in relatively better fit compared to the correlated outcomes and negative perceptions of peer events mediation models (See Supplemental Materials Table 1). Baseline adolescent heightened ∆ERN prospectively predicted increases in internalizing symptoms at 6-month follow-up (*β* = − 0.26, *SE* = 0.22, *p* = .018) and increases in internalizing symptoms at 6-month follow-up were associated with increases in negative perceptions of peer events at 6-month follow-up (*β* = − 0.40, *SE* = 0.05, *p* < .001). The direct effect of baseline adolescent ∆ERN prospectively predicting increases in negative perceptions of peer events at 6-month follow-up was not significant (*β* = 0.09, *SE* = 0.11, *p* = .349). The total model accounted for 56.90% (R^2^ = 0.569) of the variance in negative perceptions of peer events at 6- month follow-up, more than the variance accounted for in the prior model, and 57.10% (R^2^ = 0.571) of the variance in internalizing symptoms at 6-month follow-up, which was less than the total effect compared to the prior mediation model. See Table 4 for full results. The indirect effect was significant (z = 0.09, SE = 0.05, 95% CI [0.01, 0.19]). However, given the relatively better fit of the reverse mediation model compared to the other models (See Table 4), there was stronger evidence that increases in internalizing symptoms mediated the prospective association between adolescent heightened ∆ERN and increases in negative perceptions of peer events.^2^

## Discussion

The current study examined whether heightened ∆ERN—a neural marker of performance error sensitivity and defensive reactivity—predicts increases in internalizing symptoms among female adolescents, and whether these associations are mediated by increases in negative perceptions of family and peer events. We found partial support for a prospective longitudinal mediational pathway from baseline female adolescent heightened ∆ERN to increases in internalizing symptoms via increases in negative perceptions of family events. However, for the negative perceptions of peer events model, there was relatively stronger support for increases in adolescent internalizing symptoms mediating the prospective pathway from baseline adolescent heightened ∆ERN to increasing negative perceptions of peer events. Across all models, these effects held while controlling for the potential impact of total interpersonal events on negative perceptions and internalizing symptoms. Across the analyses, effect sizes were moderate in magnitude as significant portions of variance in negative perceptions of interpersonal events and internalizing symptoms were accounted for even though the sample size was modest.

Heightened ∆ERN among adolescents might have a strong impact on how adolescents interpret ambiguous family interactions. Prior to adolescence, the family context has the largest influence on youth mental health. Although peers become more influential as adolescents progress through development, family events still impact the developing child, and negative perceptions of family develop early on and likely persist through youth development. In fact, we found that there was strong stability between negative perceptions of family events across baseline and 6-month follow-up, even while controlling for the total family events adolescents experienced at each time point. Despite this stability, heightened ∆ERN still emerged as a prospective predictor of increases in negative perceptions of family events, which in turn predicted increases in internalizing symptoms while controlling for baseline internalizing symptoms. This effect was also present above and beyond the prospective association between maternal MDD history and increases in internalizing symptoms. Moreover, maternal MDD history was not correlated with, or predictive of, negative adolescent perceptions of family events at any time point. Therefore, these patterns might still generalize to family environments where mothers do not have histories of psychopathology.

Interestingly, we found stronger evidence for increases in internalizing symptoms mediating the prospective link between baseline adolescent ∆ERN and increases in negative perceptions of peer events. In fact, associations between negative perceptions of peer events and internalizing symptoms changed across the two assessment periods, becoming much more strongly related at the 6-month follow-up assessment. This might be reflective of the rapidly changing landscape of peer relationships in adolescence (e.g., Steinberg, 2000). The autoregressive paths between baseline negative perceptions of peer events and 6-month follow-up negative perceptions were not significant in any of the models. This contrasted with the strong temporal stability that negative perceptions of family events had across time. Interestingly, even though adolescents reported experiencing similar levels of peer events at each time point, adolescents’ negative perceptions of these events were greater at the 6-month follow-up compared to baseline. Furthermore, internalizing symptoms and perceptions of peer events were only significantly related to each other at the 6-month follow-up. This is consistent with prior research that has shown that even adolescents with subclinical internalizing symptoms endorse more negative subjective appraisals of interpersonal events compared to those without symptoms (Krackow & Rudolph, 2008) and is consistent with transactional developmental cascade processes among maladaptive cognitive styles, internalizing symptoms, and interpersonal relationships across adolescence (Hankin et al., 2010). Although this study cannot evaluate transactional associations among these processes due to the two-wave design, it could provide a blueprint for future studies that seek to understand how the associations among ERN, appraisals of interpersonal events, and internalizing symptoms unfold and bidirectionally influence each other in different ways over time.

This reverse mediation pattern could also be interpreted within cognitive vulnerability models of depression (Beck, 2002; Leppänen, 2006), which posit that internalizing symptoms can activate negatively biased processing of subsequent interpersonal experiences. Research shows that elevated internalizing symptoms increase adolescents’ negative interpretation of peer cues (Prinstein et al., 2005; Sandstrom et al., 2003) and erode peer relationship quality over time, generating further negative experiences (Kistner et al., 1999; Rudolph et al., 2000). However, these interpretations are speculative since this was a two-wave design where the mediator and outcome variables were measured at the same time point.

Despite study design limitations, findings could guide future preventive and intervention efforts that focus on targeting both neural sensitivity and maladaptive appraisals. Adolescents with heightened ∆ERN appear prone to interpret experiences in more negative ways, and negative feedback loops might emerge and sustain internalizing symptoms and impairments across family and peer relationships. Early preventive efforts that focus on reshaping maladaptive appraisal patterns, such as cognitive restructuring or bias modification techniques, may therefore reduce the perceived threat of interpersonal stressors. Some support for this approach comes from recent experimental work that has shown that neural sensitivity to errors can be modified and may be an important target for the prevention of mental health difficulties. For example, Meyer and colleagues (2020) demonstrated that a brief, computerized intervention targeting error sensitivity reduced ∆ERN amplitude in college students, while Klawohn and colleagues (2020) found that attentional bias modification training successfully modulated hyperactive error-monitoring in individuals with OCD. Extending beyond psychosocial strategies, converging evidence suggests that error-related monitoring processes are also pharmacologically malleable. For example, Gorka and colleagues (2018) demonstrated that adults treated with selective serotonin reuptake inhibitors (SSRIs; including sertraline, escitalopram, and paroxetine) demonstrated an increase in ERN amplitudes following 12 weeks of treatment relative to pretreatment, indicating that serotonergic modulation can alter neural response to errors. These findings suggest that medications targeting neurotransmission may complement cognitive and behavioral approaches in modifying neural risk markers associated with internalizing psychopathology.

Notably, most existing ERN-modulation studies have been conducted in adults or older adolescents (e.g., Meyer et al., 2020; Klawohn et al., 2020; Gorka et al., 2018), with limited research directly testing these approaches in youth. Given developmental differences in neural plasticity and in the salience of social and interpersonal events during early and mid-adolescence, future programs should adapt such interventions to include ecologically valid, developmentally relevant scenarios—such as peer evaluation, academic feedback, or family conflict—to enhance generalizability to real-world contexts. Moreover, evidence that heightened ∆ERN may attenuate CBT responsiveness in some individuals (Meyer, 2022) suggests that pairing cognitive approaches with methods that directly modulate neural sensitivity (e.g., mindfulness, neurofeedback, or emotion regulation training) within these salient interpersonal contexts may yield more durable reductions in internalizing symptoms.

Notably, maternal depression history remained a unique predictor of internalizing symptoms, even when accounting for ∆ERN and appraisal biases of interpersonal events. This finding highlights the potential enduring influence of familial depression risk in predicting internalizing symptoms in offspring. Maternal depression may exert its effects through genetic transmission, emotion socialization practices, or broader environmental influences, each of which may interact with adolescents’ neurocognitive vulnerabilities to shape internalizing trajectories. From a neuroscience perspective, maternal depression has been linked to alterations in offspring neural systems supporting error monitoring and affective processing (e.g., increased ∆ERN amplitude and heightened activation in limbic and salience networks; Kujawa et al., 2014; Meyer et al., 2020). These inherited or learned patterns of neural hyper-reactivity may sensitize adolescents to interpersonal stressors and contribute to the intergenerational transmission of internalizing risk. Integrating maternal depression history with adolescent ∆ERN trajectories could therefore clarify how familial and neural factors jointly confer vulnerability to maladaptive stress appraisals and affective outcomes. It is also possible that maternal depression operates as a moderator of these associations; however, the current study was underpowered to evaluate a multigroup model to determine whether effects differed depending on whether female adolescents had mothers with histories of internalizing psychopathology. Several mothers in the sample also had histories of comorbid anxiety disorders, and teasing apart whether effects differ across diagnostic subgroups is also an important area of inquiry.

Despite the study’s strengths, there were several important limitations, and findings should be interpreted cautiously. With respect to the study design, the sample size was modest, there was a significant amount of missing EEG and follow-up data, and we did not conduct an a-priori power analysis as the data was drawn from an existing data set. Although FIML is a robust estimation method that has been found to produce less biased estimates in comparison to listwise deletion (Enders, 2022; Graham, 2009; Little, 2013), results should be replicated in larger samples with adequate power and less missing data to increase confidence in the findings. In addition, the generalizability is constrained by the sampling procedures and sample characteristics. For example, the sample only included adolescents who were assigned female at birth. A couple of studies suggest that neural–social processes may operate similarly in male adolescents. For instance, in a mixed-sex adolescent sample, Niu and colleagues (2023) found that heightened ∆ERN in peer settings predicted greater fear of negative evaluation, indicating that enhanced neural reactivity to errors in social contexts may generalize across females and males. Future work should include adolescents assigned male at birth and adolescents with diverse gender identities to determine whether the associations among ∆ERN, interpersonal appraisals, and internalizing symptoms generalize to other biological sex and gender identities. Similarly, although our adolescent sample was diverse with respect to self-identified racial and ethnic identities, future investigations should examine how experiences of racial discrimination and social exclusion within peer settings that are disproportionally experienced by racial and ethnic minorities might contribute to individual differences in ERN-related threat monitoring and internalizing symptom associations. Furthermore, it will be important for future work to consider multicultural factors that shape family relationships and interactions and the role these processes might have within the context of ERN and internalizing symptom patterns.

Next, female adolescents could not have lifetime or current depressive disorders but could have histories of other internalizing psychopathologies (anxiety), and a small portion of adolescents had histories of anxiety disorders. This is an important consideration given the larger literature that has shown more consistent associations between enhanced ERN and anxiety symptoms and disorders (e.g., Olvet & Hajcak, 2008), whereas the pattern of associations between ERN and depressive symptoms has been inconsistent, with some studies indicating there is more evidence that blunted ERN is associated with depressive symptoms (Weinberg et al., 2012). Thus, our finding that enhanced ERN was associated with greater internalizing symptoms, which included anxiety and depressive symptoms, could be because some adolescents had histories of anxiety disorders and no adolescents had histories of any type of depressive disorder (e.g., persistent depressive disorder [PPD], MDD). Taken together, future work should seek to include adolescents with histories of depression as this might clarify the direction of ERN and broad band internalizing symptom associations. This would also address the limitation that the sample could in part represent a resilient population given none of the participants met lifetime or current criteria for depressive disorders at baseline. Indeed, some epidemiological data indicates that the average age of onset for depressive disorders (MDD, PPD) is 11 to 14 years old, and these rates nearly double by ages 17 and 18 years old (Lewinsohn et al., 1998; Merikangas et al., 2010). However, despite exclusion criteria for depressive disorder history, the adolescents who had mothers with histories of MDD reported significantly higher levels of internalizing symptoms compared to those whose mothers did not have any psychiatric histories at baseline; and furthermore, maternal MDD history emerged as a robust prospective predictor of increases in internalizing symptoms across all models. Taken together, adolescents in the high-risk group were likely not a resilient population given their clinical characteristic differences with the adolescents in the low-risk group at baseline and demonstrated increases in internalizing symptoms over time. In fact, research has consistently shown that even individuals with subclinical depressive and anxiety symptoms demonstrate comparable functional impairments as those who meet clinical cut-offs (Angold & Costello, 2000; Greer & Joseph, 2019).

The most significant limitation of our study is that we only had two-waves of data, and the mediators and outcomes were measured at the same time points. Although these repeated assessments are a strength for evaluating mediation, a stronger approach would have been to have multiple waves of data to allow for clearer evaluation of causal order and to formally test whether the directionality of mediating pathways shifts over development. With only two waves, reciprocal effects cannot be reliably estimated, which requires a minimum of three waves (Cole & Maxwell, 2003; Little, 2013). In balance, our approach of testing multiple competing models provides support for mediational pathways as the correlated outcomes models fit the data poorly compared to the models with directional pathways from the mediator to the outcome.

There are several important future directions for this area of research. For example, romantic relationships have been shown to contribute to changes in internalizing symptoms (La Greca & Harrison, 2005). We could not consider how baseline ∆ERN might predict perceptions of romantic relationships and, in turn, increase risk for internalizing symptoms, or vice versa, due to the low frequency of romantic events that were reported among adolescents at each time point. This is consistent with developmental trends, as by age 13, only about one-third of adolescents have been in a romantic relationship, whereas such romantic experiences and relationships become more common and intimate later in adolescence, with over 70% of 17-year-olds having experienced a romantic relationship during their lifetime (Carver et al., 2003). Given that the average age of adolescents in our sample was 13.5 years, it is not surprising that few participants reported romantic experiences. It will be important for future work to integrate other types of interpersonal relationships and contexts that are influential for mental health and adjustment. Future studies should also examine age as a moderator of these associations, as the relative influence of family versus peer contexts and the maturation of error-monitoring systems shift substantially across adolescence (Tamnes et al., 2013). Although age was not correlated with any of the variables in the current study and we did not hypothesize that the directionality of effects would differ across our sample’s age range, the magnitude of associations among ΔERN, negative appraisals of interpersonal events, and internalizing symptoms may vary as a function of developmental stage. For example, the link between ΔERN and internalizing symptoms via peer event appraisals may strengthen as adolescents progress through development and peer relationships become increasingly central to identity and emotional well-being. Future longitudinal studies that follow cohorts across a broader age span would be better positioned to test whether these pathways are amplified in older versus younger adolescents.

Finally, future studies should also test the specificity of these associations by examining whether ΔERN and negative appraisals of interpersonal events predict internalizing versus externalizing outcomes differentially. In the current sample, there was limited variability in externalizing symptoms; however, future work in mixed-risk or community samples with adequate variability in externalizing psychopathology would help clarify whether the neural and cognitive mechanisms examined here confer transdiagnostic risk or are specific to the internalizing spectrum. Although the present findings were specific to the ERN, it could be beneficial for future well-powered studies to examine other neural markers of error processing, such as the error positivity (Pe), which has also been linked to internalizing symptoms and thought to reflect conscious awareness of errors (Di Gregorio et al., 2018; Gehring et al., 2012). This work could clarify whether maladaptive appraisals of family and peer events are uniquely associated with early automatic error-processing mechanisms indexed by the ERN or extend to later stages of error processing and thereby could enhance preventive intervention precision.

In sum, by integrating a neural risk marker (ERN) with adolescents’ subjective appraisals of developmentally salient interpersonal events in a short-term longitudinal female adolescent sample, this study advances our understanding of multiple potential mediating mechanisms that contribute to longitudinal associations among ERN, maladaptive perceptions of interpersonal events, and internalizing symptoms in adolescence. Integrating neuroscientific and developmental models in this way has the potential to guide more mechanistic investigations of how neural vulnerabilities, appraisal styles, and internalizing symptoms mutually influence each other, which could help to inform more targeted and synergistic intervention approaches that could lead to more enduring improvements in mental health and interpersonal relationships.

## Supplementary Information

Supplementary Material 1.

## Data Availability

The data that support the findings of this study are available from the corresponding author upon reasonable request.

## Footnotes

1 We also tested the two mediation models with only participants with complete data (*n* = 42), and a similar pattern was found where increases in negative perceptions of family events mediated the prospective association between baseline ∆ERN and increases in internalizing symptoms.

2 We also tested and compared the two mediation models with participants with complete data (*n* = 42) and found similar coefficient estimates.
