# Supplementary material for: Exploring Longitudinal Associations Among Error-Related Negativity, Perception of Interpersonal Events, and Internalizing Symptoms in Female Adolescents
Source: Res Child Adolesc Psychopathol. 2026 Jul 15;54(4):90. doi: 10.1007/s10802-026-01485-4 (PMC13369685; doi:10.1007/s10802-026-01485-4)
Supplement: Supplementary file 1 — Supplementary Material 1. [file 10802_2026_1485_MOESM1_ESM.docx]

**Supplemental Materials**

**Supplemental Figs. 1-3. Partial Regression Scatter Plots of Baseline Adolescent ∆ERN and Internalizing Symptoms and Negative Perceptions of Interpersonal Events at 6-month follow-up while controlling for baseline assessments**


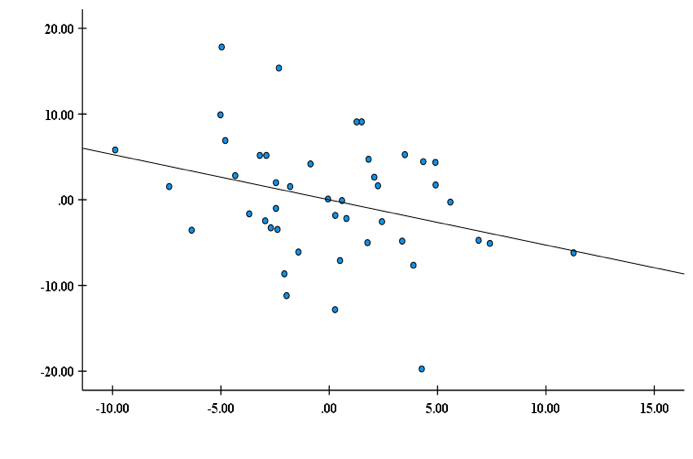


Adolescent Internalizing symptoms

at 6-month follow-up

Baseline Adolescent ∆ERN

Fig. 1. Partial regression scatter plot of baseline adolescent ∆ERN and internalizing symptoms at follow-up


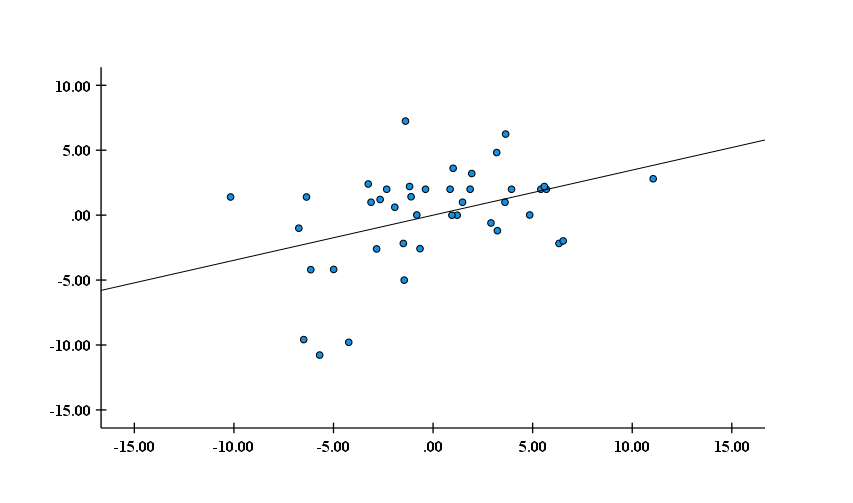


Adolescent Negative Perceptions of Family Events at 6-month follow-up

Baseline Adolescent ∆ERN

Fig. 2. Partial regression scatter plot of baseline adolescent ∆ERN

and negative perceptions of family events at follow-up


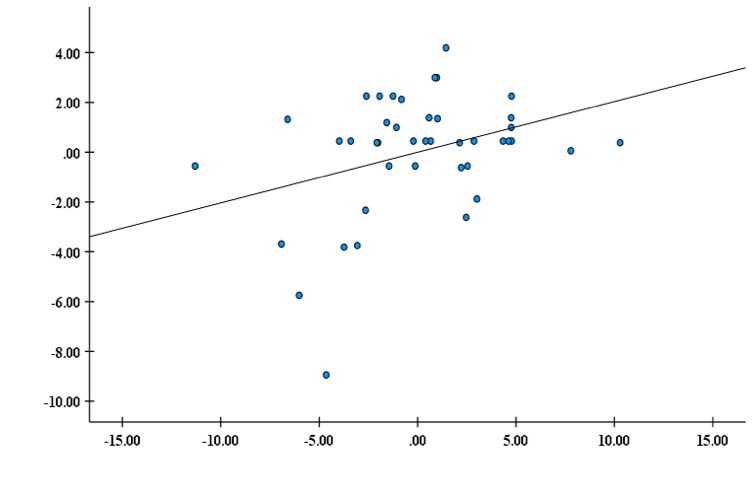


Adolescent Negative Perceptions of Peer events at 6-month follow-up

d

Baseline Adolescent ∆ERN

Fig. 3. Partial regression scatter plot of baseline adolescent ∆ERN

and negative perceptions of peer events at follow-up

**Supplemental Table 1. Model fit comparisons of negative perceptions of family events models**

| Perceptions of Peer Events Models | χ^2^ (N=79) | CFI | RMSEA | AIC |
| --- | --- | --- | --- | --- |
| Correlated Outcomes | *χ*^2^ (3) = 5.86, *p* = .119 | .98 | .11 90% CI (.00, .24) | 107.86 |
| Mediator: Negative Perceptions of Peer Events | *χ*^2^ (3) = 5.34, *p* = .105 | .98 | .10 90% CI (.00, .24) | 107.37 |
| Reverse Mediator: Internalizing Symptoms | *χ*^2^ (3) = 1.98, *p* = .576 | 1.00 | 0.0 90% CI (.00, .16) | 103.98 |

| Perceptions of Family Events Models | χ^2^ (N = 79) | CFI | RMSEA | AIC |
| --- | --- | --- | --- | --- |
| Correlated Outcomes | *χ*^2^ (3) = 10.92, *p* = .012 | .91 | .18, 90% CI (.08, .31) | 112.92 |
| Mediator: Negative Perceptions of Family Events | *χ*^2^ (3) = 3.60, *p* = .308 | 1.00 | .05, 90% CI (.00, .20) | 105.60 |
| Reverse Mediator: Internalizing Symptoms | *χ*^2^ (3) = 8.81, *p* = .032 | .96 | .16, 90% CI (.04, .28) | 110.81 |

**Supplemental Table 2. Model fit comparisons of negative perceptions of peer events models**

| **Supplemental Table 3. ∆ERN, Negative Perception of Family Events, and Internalizing Symptom Models** | | | | |
| --- | --- | --- | --- | --- |
| ***Correlated Outcomes model*** | ***β*** | ***B*** | ***SE*** | ***p*** |
| Base. intern. symptoms 🡪 intern. symptoms at 6 mos. | .52 | .62 | .10 | < .001 |
| Base. neg. percept. of family events 🡪 neg. percept. of family events at 6mos. | .71 | .67 | .10 | < .001 |
| Base. ∆ERN 🡪 neg. percept. of family events at 6 mos. | .23 | .22 | .07 | .002 |
| Base. ∆ERN 🡪 intern. symptoms at 6 mos. | -.30 | -.589 | .19 | .002 |
| Base. Total Family Events 🡪 intern. symptoms at 6 mos. | -.39 | -1.15 | .35 | .001 |
| Base. Total Family Events 🡪 neg. percept. of family events at 6 mos. | .20 | .28 | .14 | .043 |
| Total family events at 6 mos. 🡪 intern. symptoms at 6 mos. | .18 | .64 | .39 | .103 |
| Total family events at 6 mos. 🡪 neg. percept. of family events at 6 mos. | -.32 | -.55 | .15 | < .001 |
| Mat. MDD history 🡪 intern. symptoms at 6 mos. | .30 | 5.17 | 1.60 | .001 |
| Mat. MDD history 🡪 neg. percept. of family events at 6 mos. | -.02 | -.19 | .63 | .767 |
| Adolescent age 🡪 intern. symptoms at 6 mos. | .10 | .49 | .46 | .292 |
| Adolescent age 🡪 neg. percept. of family events at 6 mos. | -.07 | -.17 | .19 | .355 |
| ***Reverse Mediation Model: Internalizing Symptoms*** | ***β*** | ***B*** | ***SE*** | ***p*** |
| Base. intern. symptoms 🡪 intern. symptoms at 6 mos. | .54 | .65 | .10 | < .001 |
| Base. neg. percept. of family events 🡪 neg. percept. of family events at 6mos. | .65 | .60 | .11 | < .001 |
| Base. ∆ERN 🡪 neg. percept. of family events at 6 mos. | .17 | .15 | .07 | .036 |
| Base. ∆ERN 🡪 intern. symptoms at 6 mos. | -.29 | -.56 | .19 | .003 |
| Intern. symptoms at 6 mos. 🡪 neg. percept. of family events at 6 mos. | -.22 | -.10 | .05 | .023 |
| Base. total family events 🡪 intern. symptoms at 6 mos. | -.39 | -1.15 | .35 | .001 |
| Base. total family events 🡪 neg. percept. of family events at 6 mos. | .09 | .12 | .15 | .414 |
| Total family events at 6 mos. 🡪 intern. symptoms at 6 mos. | .18 | .63 | .39 | .111 |
| Total family events at 6 mos. 🡪 neg. percept. of family events at 6 mos. | -.28 | -.46 | .14 | .001 |
| Mat. MDD history 🡪 intern. symptoms at 6 mos. | .29 | 5.04 | 1.60 | .002 |
| Mat. MDD history 🡪 neg. percept. of family events at 6 mos. | .07 | .54 | .67 | .423 |
| Adolescent age 🡪 intern. symptoms at 6 mos. | .09 | .47 | .46 | .310 |
| Adolescent age 🡪 neg. percept. of family events at 6 mos. | -.04 | -.09 | .18 | .628 |

*Note*. Base. = Baseline. Intern. = Internalizing. Mos. = Months. ∆ERN = Error-Related Negativity Residual. Neg. = Negative. Percept. = Perceptions. Mat. = Maternal. MDD = Major Depressive Disorder.

| **Supplemental Table 4. ∆ERN, Negative Perceptions of Peer Events, and Internalizing Symptom Models** | | | | |
| --- | --- | --- | --- | --- |
| ***Correlated Outcomes Model*** | ***β*** | ***B*** | ***SE*** | ***p*** |
| Base. intern. symptoms 🡪 intern. symptoms at 6 mos. | .49 | .58 | .11 | < .001 |
| Base. neg. percept. of peer events 🡪 neg. percept. of peer events at 6 mos. | .15 | .16 | .11 | .140 |
| Base. ∆ERN 🡪 neg. percept. of peer events at 6 mos. | .25 | .21 | .09 | .022 |
| Base. ∆ERN 🡪 intern. symptoms at 6 mos. | -.28 | -.54 | .22 | .012 |
| Base. Total Peer Events 🡪 intern. symptoms at 6 mos. | -.16 | -.48 | .36 | .177 |
| Base. Total Peer Events 🡪 neg. percept. of peer events at 6 mos. | .10 | .14 | .19 | .468 |
| Total Peer events at 6 mos. 🡪 intern. symptoms at 6 mos. | -.00 | -.01 | .45 | .975 |
| Total Peer events at 6 mos. 🡪 neg. percept. of peer events at 6 mos. | -.52 | -.88 | .21 | < .001 |
| Mat. MDD history 🡪 intern. symptoms at 6 mos. | .35 | 5.94 | 1.75 | < .001 |
| Mat. MDD history 🡪 neg. percept. of peer events at 6 mos. | -.16 | -1.18 | .83 | .156 |
| Adolescent age 🡪 intern. symptoms at 6 mos. | -.05 | -.11 | .27 | .690 |
| Adolescent age 🡪 neg. percept. of peer events at 6 mos. | .10 | .52 | .55 | .350 |
| ***Mediation Model: Negative Perceptions of Peer Events*** | ***β*** | ***B*** | ***SE*** | ***p*** |
| Base. intern. symptoms 🡪 intern. symptoms at 6 mos. | .48 | .58 | .10 | < .001 |
| Base. neg. percept. of peer events 🡪 neg. percept. of peer events at 6 mos. | .18 | .19 | .12 | .097 |
| Base. ∆ERN 🡪 neg. percept. of peer events at 6 mos. | .25 | .21 | .09 | .022 |
| Base. ∆ERN 🡪 intern. symptoms at 6 mos. | -.18 | -.35 | .21 | .101 |
| Neg. percept. of peer events at 6 mos. 🡪 intern. symptoms at 6 mos. | -.37 | -.84 | .27 | .002 |
| Base. total peer events 🡪 intern. symptoms at 6 mos. | -.16 | -.47 | .33 | .152 |
| Base. total peer events 🡪 neg. percept. of peer events at 6 mos. | .12 | .16 | .18 | .391 |
| Total peer events at 6 mos. 🡪 intern. symptoms at 6 mos. | -.20 | -.76 | .48 | .110 |
| Total peer events at 6 mos. 🡪 neg. percept. of peer events at 6 mos. | -.52 | -.87 | .21 | <.001 |
| Mat. MDD history 🡪 intern. symptoms at 6 mos. | .29 | 4.97 | 1.62 | .002 |
| Mat. MDD history 🡪 neg. percept. of peer events at 6 mos. | -.16 | -1.16 | .83 | .162 |
| Adolescent age 🡪 intern. symptoms at 6 mos. | .09 | .45 | .51 | .379 |
| Adolescent age 🡪 neg. percept. of peer events at 6 mos. | -.05 | 0.11 | .27 | .689 |

*Note*. Base. = Baseline. Intern. = Internalizing. Mos. = Months. ∆ERN = Error-Related Negativity Residual. Neg. = Negative. Percept. = Perceptions. Mat. = Maternal. MDD = Major Depressive Disorder.
